# Age at First Alcohol Use as a Possible Risk Factor for Adolescent Acute Alcohol Intoxication Hospital Admission in the Netherlands

**DOI:** 10.1111/acer.14226

**Published:** 2019-11-29

**Authors:** Loes de Veld, Joris J. van Hoof, Sabine Ouwehand, Nicolaas van der Lely

**Affiliations:** ^1^ Department of Pediatrics Reinier de Graaf Gasthuis Delft The Netherlands; ^2^ Faculty of Behavioral, Management and Social Sciences Department of Communication Science University of Twente Enschede The Netherlands

**Keywords:** Adolescents, Acute Alcohol Intoxication, Age at First Alcohol Use

## Abstract

**Background:**

The primary objective of this study is to determine whether age at first alcohol use is a determinant for adolescent acute alcohol intoxication characteristics, such as age at first acute alcohol intoxication and blood alcohol concentration (BAC) at hospital admission. Around the world, as in the Netherlands, a key aim of alcohol policy is to postpone the age at first alcohol use. This is based on cohort studies that indicate a relationship between a younger age at first alcohol use and subsequent adult alcohol use disorders.

**Methods:**

This study was conducted using a cohort of data comprising individuals under 18 years of age. Data were collected between 2007 and 2017 by the Dutch Pediatric Surveillance System (NSCK) in order to monitor trends in admissions for acute alcohol intoxication. Multivariate linear regression analyses were used to determine the association between age at first alcohol use and acute alcohol intoxication characteristics, such as age at first acute alcohol intoxication and BAC at admission.

**Results:**

This study indicates that among adolescents admitted for acute alcohol intoxication, adolescents who started drinking at ≤ 14 years of age are significantly more often female, lower educated, and raised in nontraditional family structures than adolescents who started drinking between 15 and 18 years of age. Multiple linear regression analyses indicated that age at first alcohol use, corrected for covariates, significantly predicted the age at acute alcohol intoxication and BAC at admission. The association between age at first alcohol use and age at intoxication was also found to be clinically relevant.

**Conclusions:**

Although causation cannot be implied based on the results of these analyses, the results of this study suggest that interventions delaying the age at first alcohol use could be successful in increasing the average age that adolescents are admitted to the hospital for acute alcohol intoxication.

## Graphical abstract

This retrospective study, with 4,912 underage patients that were treated with acute alcohol intoxication, indicated that age at first alcohol use is significantly associated with age at admission. Patients that indicated they started drinking at a younger age are also younger when treated for alcohol intoxication and have lower blood alcohol concentration. Delaying the age of first alcohol use could not only be beneficial for a healthier lifestyle, but is also an indicator for the age of acute alcohol intoxication.

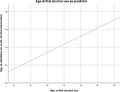

## Background

According to the World Health Organization, almost half (49.8%) of the European population between 15 and 19 years of age has used or is using alcohol (WHO, 2018). Although adolescents drink less often than adults, they consume higher quantities of alcohol per occasion (SAMHSA, 2013). Adolescent drinking has been associated with numerous negative health risks, as well as social and economic consequences (Hingson and Kenkel, 2004). In the Netherlands, prevention of adolescent drinking is considered a relevant topic, as adolescent drinking impacts not only the user but also society as a whole (Ministry of Health, Welfare and Sport, 2019; National Institute for Public Health and the Environment, 2018).

The prevention of underage drinking requires a comprehensive approach that should include but should not be limited to alcohol policy, limiting the marketing of alcoholic beverages and increasing awareness among adolescents by education (Gutierrez and Sher, 2015). Alcohol policy is based on 3 factors: reducing availability, reducing affordability, and reducing acceptability (WHO, 2014). Across the world, as in the Netherlands (VWS, 2018), a key aim of alcohol policy is to postpone the age at which alcohol is first used.

The rationale of this policy is based on cohort studies that indicate a relationship between a younger age at first alcohol use and adult alcohol use disorders (Bonomo et al., 2004; Dawson et al., 2008; Newton‐Howes et al., 2019; Rossow and Kuntsche, 2013; Von Diemen et al., 2008). The age at first alcohol use is a frequently studied risk factor not only for alcohol use disorders but also for alcohol consumption levels among adult general drinking population (Henry et al., 2011; Liang and Chikritzhs, 2013; Morean et al., 2014). The age at first alcohol use has been associated with not only negative health outcome measures such as alcohol use disorders later in life but also negative economic and social parameters, such as delinquency, poverty, and broken family structures (Morean et al., 2014).

## Research Question and Objectives

Although many cohort studies suggest a causal relationship between age at first alcohol use and adult drinking problems, a recent systematic review based on prospective follow‐up cohort studies did not provide evidence for this causal relationship (Maimaris and McCambridge, 2014). This review suggests that policy makers should concentrate on minimizing acute and short‐term harms associated with drinking among children rather than focusing on uncertain long‐term harms and suggests that more research is needed to address this relationship. The current study adheres to this recommendation by investigating whether the age of drinking onset is a risk factor for acute alcohol intoxication in adolescence. In the Netherlands, it is unknown whether age at first alcohol use is also associated with acute alcohol intoxication parameters, such as age of intoxication, BAC, and duration of reduced consciousness as a result of acute alcohol intoxication.

The primary objective of this study is to determine whether age at first alcohol use is a determinant for adolescent acute alcohol intoxication characteristics, such as age at intoxication and blood alcohol concentration (BAC) at the time of admission. This study was conducted using a cohort of data comprising individuals under 18 years of age. Data were collected between 2007 and 2017 by the Dutch Pediatric Surveillance System (NSCK) in order to monitor trends in admissions for acute alcohol intoxication.

## Materials and Methods

### Study Design and Study Population

To explore the potential relationship between age at first alcohol use and acute alcohol intoxication among Dutch adolescents, a retrospective cohort study was conducted. The study was based on a nationwide cohort of adolescents younger than 18 years of age who were treated in a pediatric department for a positive BAC. Between the years of 2007 and 2017, a total of 6,828 cases of acute alcohol intoxication were reported to the Dutch Pediatric Surveillance System. During the admission, various characteristics of the acute alcohol intoxication event, patterns of prior substance use, and social demographics were registered in the system. Cases where patterns of prior substance use were unknown, as well as cases in which the age of first alcohol use was younger than 5 years of age (*n = *1,916) were excluded from analyses, leading to 4,912 useable registrations for the current investigation. The cutoff value for age at first alcohol use was set on 5 years based on the definition of the parameter and in order to reduce the effect of outliers.

### Data Collection

Data collection had been previously performed by the Dutch Pediatric Surveillance System (NSCK). The details of data collection based on this study population have been described in several prior articles (Nienhuis et al., 2017; Van Hoof et al., 2011; Van Zanten et al., 2013). The NSCK is a nationwide surveillance system used to obtain data to support research on diagnostics, treatment, and prognosis of 10 to 12 predetermined diseases, disorders, or syndromes. In 2007, the rising trend of admissions for acute alcohol intoxication observed by pediatricians was the reason that acute alcohol intoxication was added to the system (Nienhuis et al., 2017).

All Dutch pediatric departments in the Netherlands cooperate to report cases of underage alcohol intoxication. The reports are based on data obtained by a questionnaire. The questionnaire is used to obtain data on general characteristics (e.g., age at first acute alcohol intoxication and sex), demographic characteristics, substance use patterns (e.g., age at first alcohol use, smoking, and substance use), and intoxication characteristics (e.g., BAC and duration of reduced consciousness). The questionnaire is completed by a member of the pediatric team who uses medical records, laboratory results, and self‐reported information from the patient.

### Measures

The primary measure for this specific study was the age at first alcohol use. There are various ways to define first alcohol use. Some studies define the age at first alcohol use as the first alcohol intoxication (e.g., first time drunk) (Henry et al., 2011; Rossow and Kuntsche, 2013). Other studies define the age at first alcohol use as the age of ingesting the first alcoholic unit (e.g., age at first glass) (Dawson et al., 2008; Liang and Chikritzhs, 2013; Morean et al., 2014; Newton‐Howes et al., 2019; Von Diemen et al., 2008). In the current study, the age at first alcohol use was defined as the age of drinking the first glass of alcohol.

To minimize the risk of recall bias by a too long duration between age at first alcohol use and admission for acute alcohol intoxication by telescoping forward (Prescott and Kendler, 1999), adolescents with an age of first alcohol use below the age of 5 were excluded. Furthermore, these outliers with a starting age below 5 years would interfere in the regression analysis. Although the age <5 years was chosen arbitrarily, it was based on research that indicates that the mean age of adolescents dates back to when they were 3.5 years old (Peterson et al., 2011). Research with adults suggests that people can remember childhood memories back only to about age 6 (Wells et al, 2014). A reported age of first alcohol use below the age of 5 was considered more likely to be caused by misinterpretation of the question (sip of alcohol instead of glass of alcohol) as a realistic answer.

General characteristics were measured as categorical variables: biological sex, educational level, ethnicity, family structure, and reason for hospital admission. The biological sex was defined as either male or female. The parameter educational level was based on the Dutch secondary school system consisting of 3 parallel levels: pre‐vocational education (low), senior general secondary education (middle), and pre‐university education (high). As the 3 levels are organized in a parallel manner instead of subsequently organized, educational level might act as a covariate, but not as a confounding factor is the association between age at first alcohol use and age at first intoxication. Family structure was a categorical variable consisting of 2 categories: traditional family structure and nontraditional family structure. In this study, traditional family structure was defined as a family in which the adolescent is raised by both biological parents. All other forms of families (single parent, divorced parents, blended families, foster care) were pooled to 1 category: nontraditional family structure. The most frequent reasons for hospital admission were included as categories: reduced consciousness, alcohol‐related accident, alcohol‐induced aggression, and a pooled category for all other reasons of admission.

Acute alcohol intoxication characteristics were measured as follows: age of admission in years, BAC in g/L, and duration of reduced consciousness in hours.

### Data Analyses

For all statistical analyses, SPSS for Windows (Version 25.0, IBM Corp, Armonk, NY) was used. Continuous variables are expressed as the means and standard deviations. For each continuous variable, normality was assessed using the Kolmogorov–Smirnov test. Nominal variables were expressed as frequencies (percentages) with 95% confidence intervals (CI).

The determinant of interest, age at first alcohol use, was measured in years. For the first analysis, this continuous variable was recoded into a new categorical variable consisting of 2 categories using a median split: age at first alcohol use ≤14 years and age of first alcohol use between 15 and 18 years of age. Pearson’s chi‐squared tests were used to analyze categorical variables including sex, educational level, ethnicity, family structure, and reason for admission. For numerical variables, an independent samples t‐test or a Mann–Whitney *U* test (performed on age at first acute alcohol intoxication, BAC, and duration of reduced consciousness) was performed. The significance level for all statistical tests was set to *α* = 0.05.

Multivariate linear regression analyses were used to determine whether an association existed between age at first alcohol use and acute alcohol intoxication characteristics, such as age at first acute alcohol intoxication and BAC at admission. The risk factor age at first alcohol use was identified as the independent variable, and the earlier mentioned outcome variables were dependent variables. Covariates included for this analysis were sex, educational level, ethnicity, family structure, and reason for admission. For the regression analysis on BAC, age at first acute alcohol intoxication was also included as covariate. Standardization in the linear regression model took place by the transformation of each predicted value by subtraction of the mean value, divided by the standard deviation of the predictive value (Table 3).

## Results

### Study Participants

Of the 6,828 participants in the study cohort, 4,941 participants completed the section of the survey assessing patterns of prior substance use. In order to reduce the effect of outliers, an additional 29 participants were excluded because the reported age at first alcohol use was 5 years or younger. Therefore, 4,912 participants were included in this study. In this study population, 50.4% of the adolescents started drinking at ≤ 14 years of age, while 49.6% started drinking between 15 and 18 years of age.

### General Characteristics

Baseline characteristics were analyzed for the 2 groups: age at first alcoholic drink ≤14 years and age at first alcoholic drink between 15 and 18 years of age. The results of these general characteristics are displayed in Table 1. The percentage of female patients admitted for acute alcohol intoxication was higher among the group that started drinking alcohol before the age of 15, χ^2^ (1, *N* = 4,879) = 50.44, *p* < 0.001. The educational level was higher in children who started drinking alcohol at the age of 15 than those who started drinking alcohol at an age younger than 15, χ^2^ (3, *N* = 4,596) = 12.10, *p* < 0.001. Among the group that started drinking at ≤14 years of age, the percentage of children living in nontraditional family structures was significantly higher than in the group that started drinking later, χ^2^ (1, *N* = 4,743) = 41,51, *p* < 0.001. There was no difference in reason for hospital admission, χ^2^ (3, *N* = 4,677) = 4.32, *p *= 0.23, or ethnicity, χ^2^ (1, *N* = 4,688) = 0.01, *p *= 0.91, between the 2 created research groups.

**Table 1:** General Characteristics

	Age at first alcohol use ≤14 years *n* = 2,477 (50.4%)	Age at first alcohol use between 15 and 18 years *n* = 2,435 (49.6%)	Chi‐square test results
Sex			***p* < 0.001**
% Male	48.0% (CI 46.0 to 50.0%)	58.2% (CI 56.2 to 60.2%)	
% Female	52.0% (CI 50.0 to 54.0%)	41.8% (CI 39.9 to 43.8%)	
Educational level			***p* < 0.001**
% Low and middle level	80.9% (CI 79.2 to 82.4%)	76.7% (CI 74.9 to 78.4%)	
% Higher level	19.1% (CI 17.5 to 20.8%)	23.3% (CI 21.6 to 25.1%)	
Ethnicity			*p* = 0.91
% Dutch	88.7% (CI 87.3 to 89.9%)	88.8% (CI 87.4 to 90.0%)	
% Other	11.3% (CI 10.1 to 12.7%)	11.2% (CI 10.0% to 12.6%)	
Family structure			***p* < 0.001**
% Traditional family structure	62.9% (CI 61.0 to 64.9%)	71.7% (CI 69.8 to 73.5%)	
% Nontraditional family structure	37.1% (CI 35.1 to 39.0%)	28.3% (CI 26.5 to 30.2%)	
Reason for admission			*p* = 0.23
% Reduced consciousness	89.0% (CI 87.7 to 90.2%)	87.8% (CI 86.4 to 89.1%)	
% Accident	8.0% (CI 6.9 to 9.1%)	9.4% (CI 8.2% to 10.7%)	
% Aggression	2.0% (CI 1.5 to 2.7%)	2.1% (CI 1.6 to 2.8%)	
% Other	1.1% (CI 0.7 to 1.6%)	0.7% (CI 0.4 to 1.2%)	

Bold highlights significant *p*‐values.

### Acute Alcohol Intoxication Characteristics

The acute alcohol intoxication characteristics are displayed in Table 2. A statistically significant difference (*z* = −36,86, *p* < 0.01; Mann–Whitney *U* test) was found between age of admission for acute alcohol intoxication, with adolescents who started drinking at ≤14 years of age having a lower mean age of admission (*M* = 14.8) than adolescents who started drinking between 15 and 18 years of age (*M* = 16.0). Although the absolute difference between BACs was only 1.0 g/l, statistical testing indicated that adolescents who started drinking at ≤14 years of age were admitted with a significantly lower BAC than adolescents who started drinking between 15 and 18 years of age (*z* = −5,73, *p* < 0.01; Mann–Whitney *U* test).

**Table 2:** Acute Alcohol Intoxication Characteristics

	Age at first alcoholic drink ≤ 14 years *n* = 2,486	Age at first alcoholic drink 15 to 18 years *n* = 2,422	Mann–Whitney *U* test results
Age at first alcohol intoxication (years)	14.8 (SD 1.2)	16.0 (SD 0.8)	***p* < 0.01**
Blood alcohol concentration (g/l)	1.88 (SD 0.56)	1.97 (SD 0.53)	***p* < 0.01**
Duration of reduced consciousness (hours)	3.0 (SD 2.5)	3.2 (SD 3.2)	*p* = 0.56

Bold highlights significant *p*‐values.

### Age at First Alcohol Use as Predictor

Regression analysis was used to investigate whether age at first alcoholic drink is significantly associated with the age of admission for intoxication, BAC, and duration of reduced consciousness. The results of multiple linear regression analysis are displayed in Table 3.

**Table 3:** Multiple Regression Analysis of (A) Age at First Acute Alcohol Intoxication, (B) Blood Alcohol Concentration

Variable	B Unstandardized regression coefficient	SE_B _Standard error of regression coefficient	β Standardized coefficient	*p*
(A)				
Intercept	8.896	0.203		
Age at first alcohol use	**0.468**	0.012	0.525	***p* < 0.001**
Sex	−0.278	0.030	−0.120	***p* < 0.001**
Educational level	0.025	0.037	0.009	*p* = 0.51
Ethnicity	−0.124	0.049	−0.034	***p* = 0.011**
Family structure	0.110	0.033	0.044	***p* = 0.001**
Reason for admission	0.132	0.033	0.053	***p* < 0.001**
(B)				
Intercept	1.047	0.142		
Age at first alcohol use	**0.021**	0.008	0.051	***p* = 0.008**
Sex	−0.096	0.018	−0.088	***p* < 0.001**
Educational level	0.111	0.022	0.084	***p* < 0.001**
Ethnicity	−0.051	0.028	−0.030	*p* = 0.07
Family structure	−0.011	0.019	−0.010	*p* = 0.55
Reason for admission	−0.115	0.019	−0.097	***p* < 0.001**
Age at first alcohol intoxication	0.052	0.009	0.111	***p* < 0.001**

Bold highlights significant *p*‐values.

The initial multiple regression analysis was run to explore the relationship between age at first alcohol use and age at intoxication. Age at first alcohol use, sex, family structure, and reason for admission significantly predicted age at first acute alcohol intoxication, *F*(6, 4141) = 299.53, *p* < 0.001. In this model, the slope coefficient for first alcohol use was 0.47 and statistically significant. Among adolescents admitted for acute alcohol intoxication, a 1‐year increase in age at first alcohol used was associated with a 0.47‐year increase in age at admission for acute alcohol intoxication.

A second multiple regression analysis was run to examine the association between age at first alcohol use and BAC. The model significantly predicted BAC. Although the slope coefficient for age at first alcohol use significantly predicted the BAC at admission, *F*(7, 3688) = 27.50, *p* < 0.001), the effect size was minimal.

## Discussion

### Main Results

In the Netherlands, the efforts of national policies to increase the age at first alcohol use have been successful. After 2014, when the minimum legal purchase age was increased from 16 for beverages with <15% alcohol (e.g., beer, wine), and 18 for spirits above 15% alcohol, to 18 years for all alcohol beverages, the percentage of adolescents who ever used alcohol before the age of 18 showed a declining trend (Nienhuis et al., 2017). Despite this reduction in general alcohol use among Dutch adolescents, the number of admissions for acute alcohol intoxication showed a rising trend (Nienhuis et al., 2017).

The relationship between age at first alcohol use and adolescent alcohol intoxication is unknown in the Netherlands. Age at first alcohol use is a commonly studied risk factor, and therefore, the current study extends and adds to prior research. Specifically, this study examined the relationship between the age at first alcohol use and adolescent alcohol intoxication parameters as opposed to previous studies examining outcomes in adulthood.

Our study indicates that among adolescents admitted for acute alcohol intoxication, adolescents who started drinking when they were ≤14 years of age are significantly more often female, lower educated, and raised in nontraditional family structures than adolescents who started drinking between 15 and 18 years of age.

Multiple linear regression analyses indicated that age at first alcohol use, corrected for covariates, significantly predicted age of acute alcohol intoxication and BAC at admission. The association between age at first alcohol use and age at first acute alcohol intoxication is also clinically relevant. Among adolescents admitted for acute alcohol intoxication, a 1‐year increase in age at first alcohol use was associated with a 0.47‐year increase in age at first acute alcohol intoxication.

### Limitations

One of the disadvantages of a retrospective study design is the influence of recall bias. In the follow‐up after acute alcohol intoxication, adolescents were required to recall the age at which they first used alcohol. Although self‐reported measures of alcohol use are generally considered to be reliable and valid (Del Boca and Noll, 2000; Lintonen et al., 2004), there is some evidence that the age at first alcohol use is influenced by recall bias.

The longer the time interval between the age at first alcohol use and reporting it is, the higher the risk of recall bias and telescoping forward (report a later age at first alcohol use) (Prescott and Kendler, 1999). However, the interval between the age at first alcohol use and the age at admission for acute alcohol intoxication is shorter in this study than in studies on the age at first alcohol use and the development of alcohol disorders. Furthermore, the comparison of the 2 groups as performed in this study is relevant since this bias occurred in both groups and the method of data collection was the same in both groups.

### Implications for Medical Practice

Although causation cannot be inferred based on the results of these analyses, the results of this study suggest that interventions that are successful in delaying the age at first alcohol use could be successful in increasing the average age that adolescents are admitted for acute alcohol intoxication. In adolescents admitted for acute alcohol intoxication, the group that started drinking before the age of 14 years was admitted at a younger age. Thus, aiming for abstinence from alcohol for as long as possible will increase the age at first alcohol use and the risk of young admission for acute alcohol intoxication, a finding that is consistent with studies associating a delay of first alcohol use with reduced levels of alcohol consumption later in life (Henry et al., 2011; Liang and Chikritzhs, 2013; Morean et al., 2014).

By studying differences in general characteristics between the group that started drinking alcohol before the age of 15 tears and the group that started drinking between 15 and 18, risk groups for a young starting age have been identified. Female patients, adolescents with a lower educational background, and adolescents living in a nontraditional family structure were significantly more likely to have their first alcoholic drink before the age of 15. These findings could be used to focus the trias of alcohol policy on the risk groups. For example, mass media campaigns specifically aimed at adolescents with a lower educational level might lead to a reduction of the acceptability of alcohol use among this group. Furthermore, informing parents of nontraditional family structure of the increased risk of a young starting age might lead to reducing availability by enhancing strict parental rules and availability of alcohol at home.

### Future Research

The results of this study suggest an association between age at first alcohol use and age of admission for intoxication. However, it remains unclear whether this association is based on a causal relationship or is the result of confounding factors. A currently planned longitudinal neuroimaging study examining the effects of delaying binge drinking on adolescent brain development may strengthen the hypothesis of a causal relationship (Bourque et al., 2016). Furthermore, reexamining statistics of adolescents admitted for acute alcohol intoxication in the Netherlands in 5 to 10 years might strengthen the evidence for a relation between age at first alcohol use and age at intoxication. Comparing statistics with other countries could be useful to determine which preventive strategies work best to postpone the age at first alcohol use.

## Funding Source

Supplemental support was received from “Stichting Jeugd & Alcohol,” a Dutch nonprofit organization that aims to prevent direct and indirect harm caused by alcohol usage among Dutch adolescents. The funders had no role in the design and conduct of the study.

## Conflict of Interest

The authors declare that they have no conflicts of interest.

## Financial Disclosure

All authors have no article‐relevant financial relationships to disclose.

## Author Declaration

The authors declare that this manuscript is original, has not been published before, and is not currently being considered for publication elsewhere. The authors also confirm that the manuscript has been approved by all authors and that there are no other persons who satisfied the criteria for authorship. All authors understand that the corresponding author, Joris J. van Hoof, is the sole contact for the editorial process and is responsible for communicating with other authors about the progress of the submission and revisions.

## Ethical Considerations

The data collection started in 2007 and was approved by the medical ethical commission of the Faculty of Behavioral, Management and Social Sciences of the University of Twente and the ethical board of the Reinier de Graaf Gasthuis Hospital Group. The study procedure follows the Helsinki Declaration on human subjects and testing. Informed consent was provided by all adolescents. For patients younger than 16 years of age, additional parental consent was required.
